# Just Sit Still and Pay Attention?—A Commentary

**DOI:** 10.1111/josh.12881

**Published:** 2020-02-23

**Authors:** Richard R. Rosenkranz, Caitlyn M. Neuendorf, Sara K. Rosenkranz, Kevin L. Sauer

**Affiliations:** ^1^ Department of Food, Nutrition, Dietetics & Health, Physical Activity and Nutrition Clinical Research Consortium, 1105 Sunset Ave, Room 321; Kansas State University, Manhattan, KS 66506; ^2^ Department of Food, Nutrition, Dietetics & Health, Physical Activity and Nutrition Clinical Research Consortium, 210 Justin Hall; Kansas State University, Manhattan, KS 66506; ^3^ Department of Food, Nutrition, Dietetics & Health, Physical Activity and Nutrition Clinical Research Consortium, 1105 Sunset Ave, Room 322; Kansas State University, Manhattan, KS 66506; ^4^ Department of Food, Nutrition, Dietetics & Health, 201 Justin Hall, Kansas State University, Manhattan, KS 66506

Physical activity is one of many factors that influence the growth and development of children, but only about 24% of children and adolescents aged 6 to 17 years participate in the recommended amount of 60 minutes of moderate‐to‐vigorous physical activity daily.^1^ A large body of evidence indicates that regular physical activity has numerous benefits for physical, mental, and cognitive health^2^ and insufficient physical activity can be detrimental to the overall health of young people, with increased risk of obesity, type 2 diabetes, hypertension, and other chronic diseases.^3^ Beyond chronic diseases, physical activity may help to ameliorate stress, sleep disorders, anxiety, depression, problem behavior, and may improve overall well‐being.^3^ Socially, children can develop resilience skills through physically active play and sports.^4^ They also learn to make decisions, develop intrinsic interests, regulate emotions, and solve problems.^5^

Although it is commonly understood that the musculoskeletal, metabolic, and cardiovascular bodily systems require the stimulus of physical activity to develop and function effectively, the central nervous system also benefits when children are physically active.^2^ Young people who are more physically active have a greater attention span, faster cognitive processing speed, and higher overall performance on standardized academic tests, compared to those who are not as active.^2^ Children who struggle academically in school may not necessarily need additional classroom time; it could be physical activity that they need.^6^ Maintaining focus for a 7‐hour period with minimal breaks can be challenging for younger students. In a randomized controlled study designed to evaluate the influence of classroom physical activity breaks, time‐on‐task was significantly higher in students after a 10‐minute classroom physical activity break, compared to a sedentary control group.^6^ Physical activity breaks taken throughout the school day may improve children's ability to focus on classroom tasks, possibly leading to better learning outcomes.

Unfortunately, in many schools, physical activity opportunities are often traded for additional classroom time.^7^ Although spending additional time on physical activity instead of academic subjects is often presumed to decrease student performance, a review of quasi‐experimental studies concluded that the addition of 1 hour of physical activity into the school curriculum did not negatively affect the academic achievement of students, and that additional curricular emphasis on physical education could result in better grades.^8^ Physical activity may actually improve learning, as it enhances students' ability to understand, retain, and apply the information they are taught.^9^ In a meta‐analysis of 26 studies of the relationship between physical activity and test scores, results showed significant, positive correlations between activity and academic performance for language, math, reading, and on‐task behavior.^10^ Furthermore, physical activity is positively associated with years of college education and overall higher grades throughout the duration of formal schooling.^11^

Physical activity has been largely engineered out of the daily lives of children through advancements in technology and other societal influences. Outside school, children often have continual access to video games, television, cellular phones, and other devices, spending around 5‐7 hours/day on a digital screen.^12^ Because children spend a large proportion of their waking hours in attendance, schools are in a unique position to help or hinder opportunities for physical activity. Students have potential opportunities to obtain school‐related physical activity in several contexts: physical education, recess, classroom activities, before or after school activities, and via active transportation to or from school.^5^ Thus, there are several contexts where opportunities could be developed, improved, or better leveraged for the promotion of children's physical activity.

Prioritizing academic emphasis over physical activity is understandable—given schools' educational mission—but what seems far worse is the purposeful withholding of physical activity, often done for behavioral management purposes.^13^ The American Academy of Pediatrics has actively discouraged withholding physical activity; nevertheless, school personnel frequently restrict or withhold recess or other active opportunities.^13^, ^14^ In 2006, researchers found that more than 80% of elementary schools allowed the restriction of recess, purportedly to strengthen academic performance or classroom management.^15^ Almost 80% of principals reported that their schools continue to take recess away from students as a form of discipline for undesirable behavior,^1^ and children who lose the opportunity to be physically active during school are unlikely to compensate for that loss after school.^16^

When it comes to other health promoting behaviors, educators and administrators recognize the importance of providing students with nutritious meals to accompany learning. They admit that without proper nourishment, it is very difficult for children to be academically successful. Although the practice of withholding lunch for behavior management purposes or pressure to achieve high test scores would never be tolerated, withholding physical activity has been allowed, despite clearly being necessary and beneficial for children. Recess, for instance, provides a context important not only for children to be physically active, but also to learn physical, social, and emotional skills.^17^ When children lose their recess or free play opportunities, children lose opportunities for physical activity, self‐expression, creative play, and are deprived of the ability to develop important skills necessary for adulthood.^7^, ^18^ Withholding, restricting, or imposing excessive limits on physical activity deserve serious scrutiny, because failure to obtain adequate amounts of physical activity—and to let kids spend time playing as they learn—has the potential to hinder the overall development, health, and academic success of children. Physical activity is an important component of the school day and helping children to reach their full potential—cognitively, physically, socially, and emotionally—should be prized.

Policy and law may be helping to change the landscape for physical activity in schools. In 2015, the No Child Left Behind Act was replaced by the Every Student Succeeds Act,^19^ which holds schools accountable for students' academic performances. For the first time, however, the definition of a well‐rounded education also included health and physical education.^19^ Since the implementation of the Healthy, Hunger‐Free Kids Act of 2010, schools have been tasked with making improvements to wellness policies that deal not only with nutritional standards and practices, but also with physical activity.^20^ Although many school districts have struggled to comply with this law, implementation frameworks such as School Wellness Integration Targeting Child Health (SWITCH) can facilitate the process for school wellness leaders to deliver evidence‐based interventions to promote physical activity and other wellness components.^21^

Beyond policy, the educational culture seems to be shifting toward an appreciation of physical activity, and there has been a recent focus on what is termed “physical literacy,” defined as maximizing individual attributes and the development of the whole child, physically and psychologically, to lead a physically active and healthy lifestyle.^22^ Physical literacy takes into account the influence of school curriculum, home environments, and community settings. A comprehensive approach that embraces physical literacy is vital to the development of physically active children, and its advocates suggest that educators and parents come together to create a physically literate culture that makes physically active lifestyles the norm.^22^ Learning how and why movement is beneficial helps to establish the purposeful and meaningful connections needed to facilitate the development of skills and capabilities to incorporate physical activity into young students' lives.

Physical activity is a vital component within the lives of children, and vital to a comprehensive, well‐rounded education. Physical activity has the ability to impact children's academics, life‐long health, and overall well‐being, and schools have opportunity to contribute positively to the physical activity needs of children without compromising their educational mandate. According to the most recent US report card on physical activity for children and youth, American schools received a grade of D‐ on physical activity support, as only 20–26% of schools are currently excelling by creating active school policies, offering quality physical education, and taking advantage of physical activity opportunities.^1^

The guiding question is: “What can schools do?” The Comprehensive School Physical Activity Program offers a free resource, developed by the Centers for Disease Control and Prevention,^23^ that helps schools develop and adopt a multi‐component approach to instill the knowledge, skills, and confidence in students to be physically active for a lifetime. This model encourages schools to create or take advantage of existing opportunities for students to be physically active. Schools that adopt a Comprehensive School Physical Activity Program make it more possible for students to achieve the nationally recommended 60 minutes of moderate‐to‐vigorous physical activity each day,^3^ of which at least 30 minutes are recommended to be obtained during the school day.^2^ Physical education classes make important contributions, but most classes are likely to provide about 10‐20 minutes of moderate‐to‐vigorous physical activity,^2^ leaving roughly half of the recommended 30 minutes to be obtained during other parts of the school day—such as during recess, classroom activities, before or after school activities, and via active transportation to or from school. In accordance with Comprehensive School Physical Activity Program, school personnel can consider several of the following recommendations for promoting physical activity.

Here are 8 specific implications for school health, with suggestions to promote children's physical activity in schools. (1) Develop school and school district policies that prohibit using exercise or physical activity as punishment, using restriction or withholding of recess as a tactic for behavior management. School districts should ensure administrator support and provide teacher training around these policies, including alternative tactics for behavior management. (2) Build physical activity breaks into classroom lessons, using resources such as online videos available through GoNoodle (https://www.gonoodle.com/) or Brain Breaks (http://www.unicefkidpower.org/brain-breaks-for-kids/). These should be easy to implement, integrated into academic concepts, and last about 5 minutes or less.^24^ (3) Develop or advocate for policies that ensure children receive 150 or more minutes of weekly physical education during school. (4) Promote active transportation through bike‐to‐school days, walking school buses,^25^ or through supportive policies and infrastructure. School‐community partnerships can develop and implement interventions to replace automobile transportation with walking or biking to school, and promote safety. Infrastructure development (such as additional sidewalks or cross walks), or recruitment of volunteers to lead active transportation, such as a walking school bus. (5) Assign physically active homework where appropriate. (6) Allocate 20 or more minutes of daily recess during the school day. For inclement weather, school staff should develop indoor recess plans that are nearly as conducive to physical activity as outdoor recess. (7) Add sessions of physical activity into before‐school or after‐school programming. Staff should consider options such as supervised free play, simple structured games, or opportunities to sample various sports. (8) Offer intramural sports or running and walking clubs, including student family member participation also.

In addition to the above suggestions, schools should work to improve the quality of offerings by providing SAAFE^26^ physical activity programs, policies, and practices. SAAFE stands for supportive, active, autonomous, fair, and enjoyable.^26^ Physical activity opportunities should be supportive of children's psychological needs, as well as active, with more movement, and less sitting or standing. Opportunities should be autonomous, allowing children to make some of their own choices. They should be fair, so that all types of kids have opportunity to participate. Last, they should be enjoyable, so physical activity is not used as punishment or to socially isolate children.

In conclusion, students in school should not passively “just sit still and pay attention.” Placing value and emphasis on physical activity can bolster schools' healthy norms, creating a supportive culture for student success and better quality of life for all.^27^ As adult decision makers, we all have a role to play in bringing up the poor (D‐) grade that our schools have earned for physical activity support, as we endeavor to help children succeed academically, physically, socially, and emotionally, now and well into the future.
